# Microwell Fluoride Screen for Chemical, Enzymatic, and Cellular Reactions Reveals Latent Microbial Defluorination Capacity for −CF_3_ Groups

**DOI:** 10.1128/aem.00288-22

**Published:** 2022-06-21

**Authors:** Madison D. Bygd, Kelly G. Aukema, Jack E. Richman, Lawrence P. Wackett

**Affiliations:** a Microbial Engineering, University of Minnesota, Minneapolis, Minnesota, USA; b Biotechnology Institute, University of Minnesota, Minneapolis, Minnesota, USA; c Biochemistry, Molecular Biology and Biophysics, University of Minnesota, Minneapolis, Minnesota, USA; Novo Nordisk Foundation Center for Biosustainability

**Keywords:** fluoride, screening, organofluorine, PFAS, defluorination, *Pseudomonas putida* F1, bacteria, trifluoromethyl, high throughput

## Abstract

The capacity to defluorinate polyfluorinated organic compounds is a rare phenotype in microbes but is increasingly considered important for maintaining the environment. New discoveries will be greatly facilitated by the ability to screen many natural and engineered microbes in a combinatorial manner against large numbers of fluorinated compounds simultaneously. Here, we describe a low-volume, high-throughput screening method to determine defluorination capacity of microbes and their enzymes. The method is based on selective binding of fluoride to a lanthanum chelate complex that gives a purple-colored product. It was miniaturized to determine biodefluorination in 96-well microtiter plates by visual inspection or robotic handling and spectrophotometry. Chemicals commonly used in microbiological studies were examined to define usable buffers and reagents. Base-catalyzed, purified enzyme and whole-cell defluorination reactions were demonstrated with fluoroatrazine and showed correspondence between the microtiter assay and a fluoride electrode. For discovering new defluorination reactions and mechanisms, a chemical library of 63 fluorinated compounds was screened *in vivo* with Pseudomonas putida F1 in microtiter well plates. These data were also calibrated against a fluoride electrode. Our new method revealed 21 new compounds undergoing defluorination. A compound with four fluorine substituents, 4-fluorobenzotrifluoride, was shown to undergo defluorination to the greatest extent. The mechanism of its defluorination was studied to reveal a latent microbial propensity to defluorinate trifluoromethylphenyl groups, a moiety that is commonly incorporated into numerous pharmaceutical and agricultural chemicals.

**IMPORTANCE** Thousands of organofluorine chemicals are known, and a number are considered to be persistent and toxic environmental pollutants. Environmental bioremediation methods are avidly being sought, but few bacteria biodegrade fluorinated chemicals. To find new organofluoride biodegradation, a rapid screening method was developed. The method is versatile, monitoring chemical, enzymatic, and whole-cell biodegradation. Biodegradation of organofluorine compounds invariably releases fluoride anions, which was sensitively detected. Our method uncovered 21 new microbial defluorination reactions. A general mechanism was delineated for the biodegradation of trifluoromethylphenyl groups that are increasingly being used in drugs and pesticides.

## INTRODUCTION

Fluorinated, particularly polyfluorinated, compounds are major pollutants and are widely considered to be poorly, if at all, biodegraded (1–3). More than one million fluorinated compounds (organic and inorganic) are recorded on PubChem (4). A subset of organofluorine compounds are categorically known as per- and polyfluoroalkyl substances (PFAS), which consist of several thousand compounds introduced into the global market (5). Current understanding of the number of commercially relevant PFAS is on the order of hundreds (6). Further, greater than 20% of newly introduced agricultural chemicals and drugs are fluorinated (7–10). In contrast, there are only dozens of known natural product organofluorine compounds (11, 12). In this context, billions of years of microbial evolution have progressed largely in the absence of fluorinated compounds. Additionally, organofluorine compounds have been introduced commercially principally because of their chemical inertness (13). For these reasons, some organofluorine compounds are considered recalcitrant due to a lack of or limited biodegradation in the environment (14).

A limited number of bacterial genes and enzymes have been demonstrated to participate in biodefluorination. The most well-studied case of biodefluorination is with a monofluorinated natural product produced by some plants and bacteria, fluoroacetate (11, 15). Fluoroacetate dehalogenase has been studied structurally and mechanistically (16–18). More recently, another structurally determined enzyme, toluene dioxygenase (Tod), was shown to be responsible for the defluorination of a difluorinated ether substrate (19). In another example, the enzyme benzoyl-coenzyme A (CoA) reductase, was shown to participate in the defluorination of 4-fluorobenzoate (20). A limited number of biodegradation studies have also been conducted with mixed and pure cultures of bacteria, including the biodegradation of heavily fluorinated compounds. Recent studies also report the biodegradation of perfluoroalkyl acids (21, 22).

Documentation of microbial degradation of fluorinated compounds typically requires monitoring parent compound disappearance and product appearance. Detecting defluorinated organic products often involves extractions followed by liquid or gas chromatography and mass spectrometry (23, 24). With polyfluorinated compounds, these analytical methods can be difficult due to extraction losses, volatility during workup, and external contamination. In addition to the many different organic products expected from the thousands of organofluorine compounds, fluoride anions are a universal coproduct of defluorination. This is due to the high electronegativity of fluorine, and fluoride is observed regardless of the mechanism of C-F bond cleavage (25, 26). Fluoride determination as a means of monitoring defluorination reactions is also ideal because natural waters and laboratory buffers typically contain very low levels of fluoride (27). This contrasts with chloride, which has a high background and thus is not an ideal indicator for dechlorination (28).

The value of fluoride anion monitoring has been recognized and has been used in single endpoint determinations in batch cultures of microbes tested for biodegradation of C-F compounds. In these studies, the two commonly used methods are ion chromatography and fluoride-specific electrode determination (21, 22, 29, 30). Each method is done via single-sample, fixed time point determinations, and only a limited number can be carried out per hour. Research on microbial defluorination would accelerate significantly with the introduction of methods to screen multiple organisms, enzymes, and libraries of fluorinated compounds rapidly and combinatorially in small liquid volumes.

Chemists have devised colorimetric and fluorimetric methods for determining and quantifying fluoride but none have been demonstrated for microbiological screening, with the exception of a colorimetric test suitable for petri plate screening of colonies showing fluoroacetate dehalogenase activity (31). That method, using xylenol orange, required an overlay of reagents due to medium interference precluding direct incorporation, and it was not demonstrated for liquids. A variety of other approaches have been developed that are functional only in organic solvents, are nonbiocompatible, and/or require extensive organic synthesis to make components that are not commercially available (32–34). As a result, the methods have not been widely adopted for studies of biodefluorination.

In the present work, we systematized a high-throughput screening protocol distinct from ion chromatography or fluoride electrode and demonstrated the method’s efficacy to rapidly monitor chemical, enzymatic, and *in vivo* microorganismal defluorination reactions. The method is based on a previously described fluoride binding reaction and uses all commercially available components (35–37). The method is sensitive; several nanomoles of fluoride are sufficient to bind to a rare earth metal coordination complex to produce a blue chromophore. For the first time, here the method was adapted for biological purposes, miniaturized, and calibrated in microtiter 96-well plates to determine fluoride concentration visually or via a spectrophotometric plate reader. The method was demonstrated with purified enzyme and cell-based defluorination reactions. Parameters of the microliter screen were determined: sensitivity, accuracy, and interfering substances. The new assay was subsequently used to screen a model microorganism against a library of 63 organofluorine compounds. Surprisingly, 21 compounds, most with multiple fluorine substituents, showed microbially catalyzed defluorination. One compound containing a –CF_3_ group underwent significant defluorination, and the mechanism of defluorination was demonstrated. Overall, this method can increase the pace of discovering new microbes and enzymes that biodegrade C-F compounds.

## RESULTS

### Standard fluoride screen parameters.

**FIG 1:**
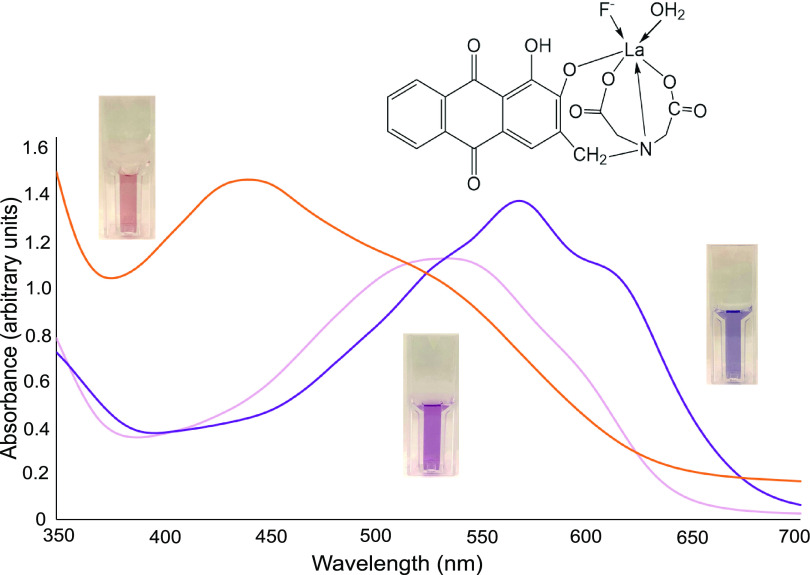
UV-Vis spectra of the lanthanum-alizarin complex bound to phosphate (orange), water (red), and fluoride (purple). The cuvettes show how the orange, red, and purple species can be differentiated visually. The upper right diagram shows the putative structure of the lanthanum-alizarin complex with bound fluoride as previously reported in several studies (34–36).

Failing to find literature on small-volume, rapid screening methods for monitoring biodefluorination, we sought here to adapt a method previously used for determining fluoride in liter volumes of drinking water. The method is based on a color change upon fluoride binding to a rare earth metal bound in complex with an alizarin ligand (Fig. 1). Based on previous work (35, 36), we tested the rare earth metals cerium, lanthanum, neodymium, and praseodymium with standard fluoride concentrations. We found lanthanum best for working in biologically relevant fluids such as buffers and growth media. Additional testing was done to determine optimal pH and concentration of the acetate buffer as well as reagent ratios. Consistent with one previous report (36), maintaining a 1:1 ratio of alizarin to lanthanum and the buffer pH in the range of 5.0 to 5.5 was found to be most sensitive for detecting fluoride. However, we determined that increasing the concentration of sodium acetate buffer 3-fold over that used previously (36) was necessary to counterbalance the buffering capacity of typical enzyme and microbiological media. All subsequent experiments described here use lanthanum as the metal, 84 mM acetate buffer at pH 5.2, and a 1:1 ratio of alizarin to lanthanum. The organic solvent, acetone, was also optimized and included routinely based on previous studies showing acetone enhanced the sensitivity of the assay (36).

The cuvette with red liquid shown in the middle of Fig. 1 represents the color of the alizarin-lanthanum-water complex, consistent with previous reports (35, 36). As fluoride is added to the medium, the absorbance maximum shifts to a peak at 565 nm with a strong shoulder at 620 nm. The increase in absorbance at the longer wavelength, 620 nm, is proportional to the amount of fluoride in the range of 10 to 100 μM (see Fig. S1 in the supplemental material). Used in shallow-well microtiter plates, the method can be monitored visually, or with a plate reader, to detect as little as 4 nmol fluoride in a 200-μL volume.

It was initially curious to us as to why this facile and sensitive method has not been reported to screen biodefluorination previously, but then we found significant interference by typical microbiological and enzymatic reagents. For example, phosphate buffer, often used at millimolar concentrations, outcompetes fluoride to make an orange product (Fig. 1). Subsequently, a series of other buffers and media (bicine, morpholinepropanesulfonic acid [MOPS], Tris, and nutrient broth) also showed significant interference (Table 1 and Fig. S2), but HEPES buffer showed little interference and provided excellent buffering for several microbial media and enzyme buffer. All subsequent experiments were conducted using HEPES as the buffering agent.

**TABLE 1:** Interfering and noninterfering buffers and growth medium components^*a*^

Buffer	Component
Noninterfering	25% acetone
	84 mM acetate buffer
	20 mM HEPES
	10% glycerol
	100 mM sodium chloride
	2 mM sodium bromide
	2 mM sodium sulfate
	2 mM sodium nitrate
	100 μM sodium phosphate monobasic
	47 μM borate (borax)
	10 mM sodium sulfide
	43 μM EDTA
	2 mM fluorobenzene
	0.5% Casamino acids
	2 mM succinic acid
	2 mM sodium acetate
	20 mM magnesium chloride
	23 mM calcium chloride
	20 mM lithium chloride
	19 mM ammonium chloride
	20 mM potassium chloride
	20% DMF
	20% acetonitrile
	2% glucose
	2% arabinose
	2% sucrose
	74 μM thiamine hydrochloride
	0.41 mM nicotinic acid
	2 μM biotin
Moderately interfering	0.2 M Tris
	0.25 M bicine
	0.2 M MOPS
	2 mM sodium phosphate monobasic
	2 mM ferric chloride
	20% ethanol
Strongly interfering	500 μM imidazole
	13 g/liter nutrient broth
	10 mM sodium phosphate monobasic
	500 μM mercaptoethanol
	0.1% yeast extract
	1.0% yeast extract
	37 g/liter brain heart infusion
	50 μM zinc chloride
	50 μM cupric chloride
	2 mM sodium citrate dihydrate

a Conditions for testing were as described in Materials and Methods. DMF, N,N-dimethylformamide.

**FIG 2:**
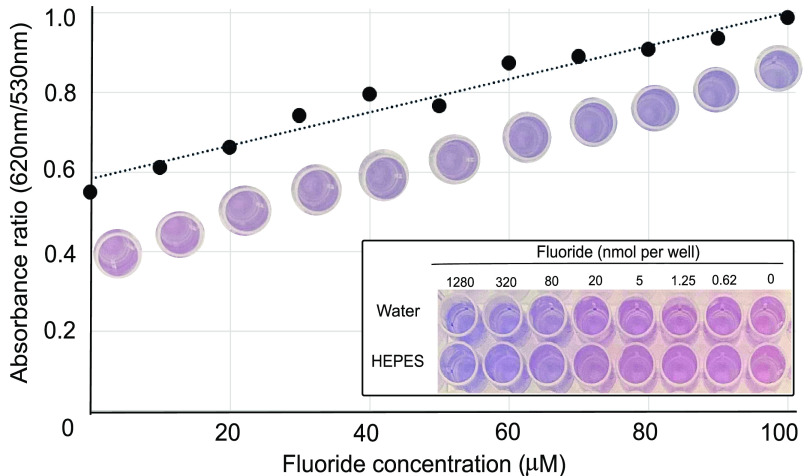
Plotted is the ratio of the lambda maximum of fluoride-bound complex (620 nm) over the lambda maximum of the unbound complex (530 nm). The 530-nm absorbance does not change during the increasing 620-nm absorbance, so the 530-nm absorbance is used as a fixed point to correct for minor baseline fluctuations. This trend of 620-nm/530-nm absorbance is linear from 0 to 100 μM fluoride, which also can be seen by eye in the assay. We define a dark purple well as having a 620/530 ratio of >0.7 and a light purple well as having a ratio of <0.7. (Inset) Comparison of fluoride detection in water versus 20 mM HEPES buffer down to a 0.62 nmol concentration; 20 mM HEPES does not inhibit the ability to detect fluoride compared to water.

Initially, the screen was calibrated with known concentrations of fluoride in HEPES buffer in microtiter well plates. As fluoride was varied, an isosbestic point was observed at 530 nm. Spectrophotometric readings were obtained by dividing the measured 620-nm absorbance by that at the constant 530-nm absorbance. That ratio plotted against fluoride concentration gave an increasing and linear response to fluoride up to 100 μM (Fig. 2) with an *R*^2^ of 0.96. The screen was routinely used in the 10 to 100 μM range, corresponding to 2 to 20 nmol fluoride in the wells. The change in color from red to purple was clearly discernible to the eye and for a spectrophotometric plate reader at and above 4 nmol fluoride in the well. In many experiments, we found visual inspection followed by photography with a simple cell phone camera to serve our purposes well. The high-throughput application of this screen can cover thousands of individual wells daily. Digital photography and available storage systems also allow for image processing that can enhance color for sensitivity, although this was not done routinely here. The method also lends itself to be used in an ultrahigh-throughput format if using robotic liquid and plate handling and spectroscopy.

### Broad testing for noninterfering and interfering biological reagents.

HEPES buffer can be used for the growth of many bacteria and for working with many enzymes *in vitro* (38, 39), but some studies will require alternative or additive compounds. In that context, a wide variety of bacterial and enzyme medium components were tested here and classified as (i) noninterfering, (ii) moderately interfering, and (iii) strongly interfering, as defined in Materials and Methods (Table 1). The noninterfering components included organic solvents, buffers, salts, sugars, vitamins, and the general growth medium Casamino Acids. These data illustrate that this method can successfully determine fluoride in the presence of components used in a wide variety of microbiological and enzyme-based experiments.

### Monitoring defluorination reactions *in vitro*.

**FIG 3:**
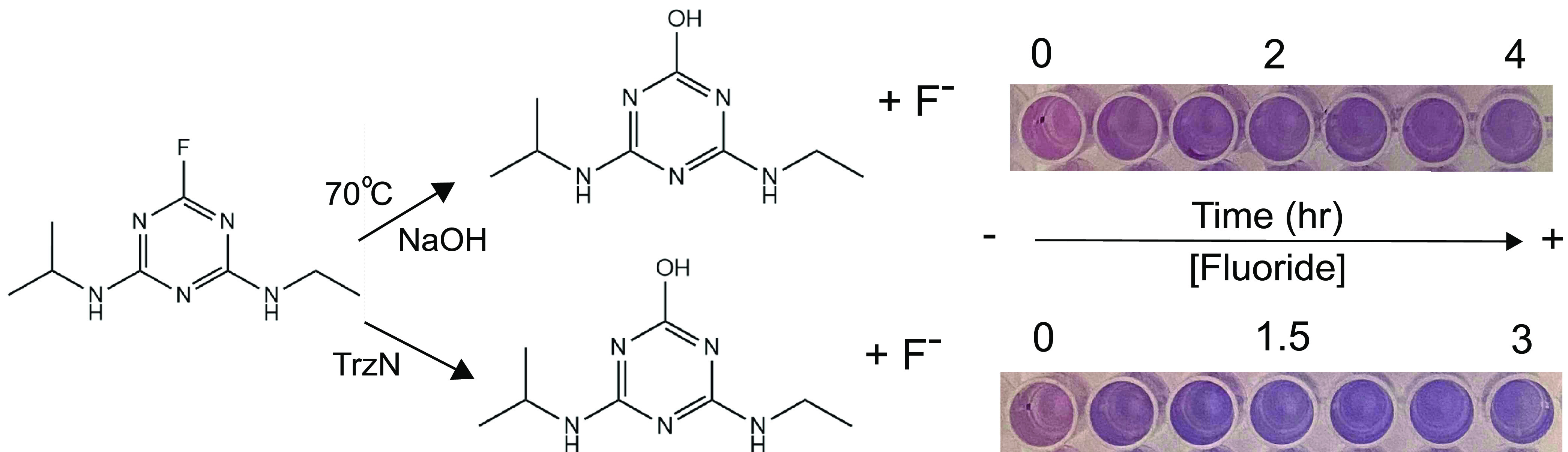
Chemical and enzymatic defluorination of fluoroatrazine catalyzed by base or triazine hydrolase (TrzN). The assay wells correspond to fluoride detection over time. Wells shown are a 1:4 dilution of the original sample.

To test the microwell method for monitoring the progress of chemical and enzymatic defluorination, we examined a defluorination reaction that could be catalyzed by either sodium hydroxide or the enzyme TrzN. The *s*-triazine ring is sufficiently activated for nucleophilic aromatic substitution, which allows for defluorination of fluoroatrazine to be elicited with sodium hydroxide (pH 11) and heat. The enzyme TrzN was previously shown to produce hydroxyatrazine from fluoroatrazine (40). Reaction time course experiments were monitored by the microwell colorimetric method (Fig. 3). This showed that fluoride was increasing over time, as expected for catalytic chemical and enzymatic reactions. Moreover, we found that we could obtain estimates of fluoride at concentrations exceeding the ideal range of color formation by making a series of 2-fold dilutions (Fig. S3). This takes advantage of the ability of this method to easily make 96 determinations at once. The results obtained with the color method were confirmed using a fluoride electrode.

### Fluoride release from cells and determination in media.

An additional issue addressed here was whether enzyme activity *in vivo* reasonably reflects the activity of the enzyme releasing fluoride internally. TrzN is known to be in the cytoplasm when recombinantly expressed in Escherichia coli (pTrzN) (41). To determine if fluoride concentration in the culture supernatant reflects fluoride release by the cytoplasmic enzyme, TrzN was expressed in whole cells of E. coli and incubated with fluoroatrazine *in vivo*, and fluoride was measured by fluoride electrode. The medium concentration of fluoride was 36 μM. An additional sample was taken at the same time, but the cells were lysed using heat, and the fluoride concentration was detected to be 40 μM. This indicated that 90% of the fluoride released by TrzN could be detected in the medium alone. That observation supported the idea that fluoride was not bound to cells in the cell pellet, and there is no need to lyse cells to release the fluoride. Avoiding cell lysis allows the assay to be high throughput and mitigates potential interferences by cytoplasmic cellular compounds. Consistent with these findings, E. coli is known to produce a highly active fluoride export protein to protect itself from fluoride, a toxic anion to bacteria (42, 43). Pseudomonas strains are also known to readily export fluoride into medium during biodegradation (44) and following external exposure (45). The data here and published literature together suggest that fluoride determination in microbiological medium will serve to detect fluoride produced by cytoplasmic enzymes.

### Screening for discovery of new defluorination reactions.

It had been known that the TrzN enzyme would defluorinate fluoroatrazine, providing a good test of the method, but the major incentive for devising a microwell screening method was to expand the known constellation of microbial defluorination reactions. In that context, we screened a library of fluorinated compounds with Pseudomonas putida F1, a model bacterium recently reported to catalyze a novel defluorination reaction with 2,2-difluoro-1,3-benzodioxole (DFBD) (19). All results from the color screening in 200 μL were compared to fluoride electrode measurements.

The screen was run with toluene-grown whole-cell suspensions of P. putida F1 incubated with 63 different fluorinated compounds in 96-deep-well plates. Growth on toluene is known to induce enzymes of the Tod pathway for the oxidation of aromatic hydrocarbons. In the screening, more than 60% of the fluorinated compounds were polyfluorinated, and a remarkable 33%, or 21 compounds, showed evidence of defluorination (Table 2 and Fig. S4). All reported compounds were chemically stable and did not undergo defluorination in the absence of microbial cells. A sample of the culture in each well was subsequently tested by fluoride electrode (Fig. S5). A side-by-side comparison was made to determine the sensitivity and selectivity of the color screen (Table 3 and Fig. S6).

**TABLE 2:**
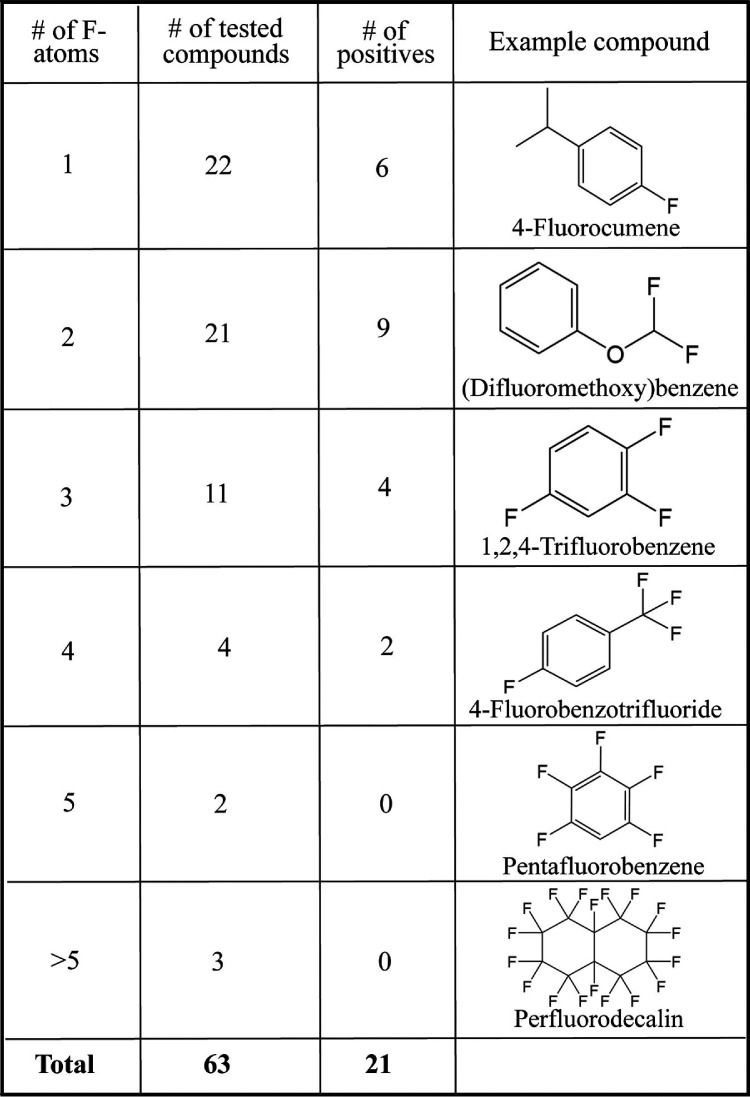
Types and number of fluorinated compounds tested, with the corresponding positive hits of fluoride release after incubation with P. putida F1 and an example of compound containing a given number of fluorine atoms

**TABLE 3:** Summarized results after 2 mM fluorinated chemical incubation with P. putida F1^*a*^

Compound	Color screen (+/−)	Electrode >30 μM (+/−)	Result
2-Fluorotoluene	+	+	True +
3-Fluorotoluene	++	+	True +
4-Fluorotoluene	+	+	True +
4-Fluorobenzotrifluoride	++	+	True +
α,α,α-Trifluorotoluene	++	+	True +
2,5-Difluorotoluene	++	+	True +
3,5-Difluorotoluene	++	+	True +
2,3-Difluorotoluene	+	+	True +
2,4-Difluorotoluene	+	+	True +
2,4,5-Trifluorotoluene	++	+	True +
1,2-Difluorobenzene	++	+	True +
1,3-Difluorobenzene	++	+	True +
1,2,3-Trifluorobenzene	++	+	True +
1,2,4-Trifluorobenzene	++	+	True +
1,3,5-Trifluorobenzene	−	+	False −
1,2,3,4-Tetrafluorobenzene	−	+	False −
1,2,3,5-Tetrafluorobenzene	+	−	False +
1,2,4,5-Tetrafluorobenzene	+	+	True +
(Difluoromethoxy)benzene	++	+	True +
Phenyl trifluoromethyl sulfide	+	−	False +
4-(Difluoromethoxy)benzoic acid	++	+	True +
4-Fluoro acetophenone	++	+	True +
2-Fluoro-4-(propanyl)benzoic acid	+	+	True +
4-Fluorocumene	+	+	True +
4-(Difluoromethoxy)aniline	+	+	True +

a Cell supernatant was tested in the color screen and measured with the fluoride electrode to confirm each positive or negative result. The cutoff for fluoride detection with the electrode was set at 30 μM, as this value was deemed to be the reliable limit of detection in the colorimetric method. If the sample in the color assay was + or ++ (see the section on screen development in Materials and Methods) and the probe reading was above 30 μM, it was considered a true positive. If the well was not purple but more than 30 μM was detected with the fluoride electrode, it was considered a false negative. If the color screen indicated purple but the probe read less than 30 μM, then it was considered a false positive.

All compounds showing a strong color reaction were positive in releasing fluoride, as determined by the fluoride electrode, a 100% selectivity by this measure. Two remaining weaker positives (lighter purple) proved to be negative in the fluoride electrode test. Two compounds positive in the fluoride electrode test were negative by visual inspection of the color screen. The few false positives and negatives were with compounds showing marginal or no defluorination. In total the method proved to be very reliable in giving true positives when fluoride release was greater than 15 nmol per microtiter well. This makes the rapid method described here reasonable for screening a directed evolution, or other large cell library, in which using the fluoride electrode would be too cumbersome. In the present study, we decided to follow up with the strongest positive to determine the mechanism of defluorination, as that was not immediately obvious.

### Unexpected insights on defluorination.

To our knowledge, 4-fluorobenzotrifluoride biodefluorination had not been studied previously, and substantial fluoride release was observed here despite the known resistance of −CF_3_ groups to biodegradation. CF_3_ groups are often substituted for CH_3_ in drugs and agricultural chemicals to inhibit biodegradation (7–17, 46). Moreover, toluene dioxygenase is known to oxidize the 2,3-carbon atoms between 1,4-substituents on an aromatic ring, making it unlikely to be directly attacking the fluorinated carbon *para-* to the CF_3_ group (47, 48). In that context, we sought here to further confirm defluorination and elucidate the mechanism by which defluorination occurs.

**FIG 4:**
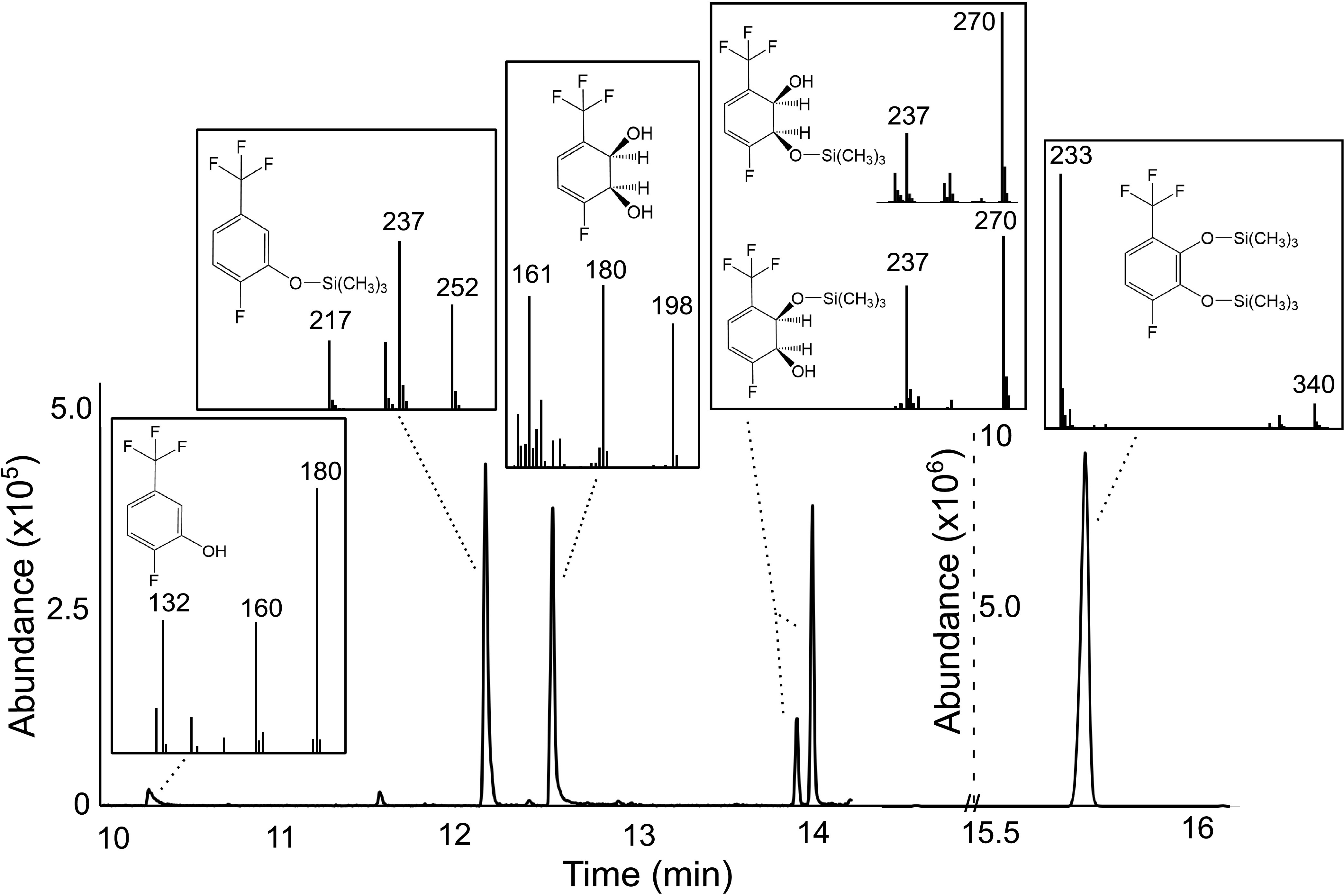
Gas chromatogram and mass spectra showing the initial fluorinated products from the oxidation of 4-fluorobenzotrifluoride by P. putida F1 and following a short-time derivatization with bis-trimethylsilane. Both derivatized and underivatized products are shown. The GC abundance for products eluting after 15.5 min is 20-fold higher, indicating that the derivatized catechol of 4-fluorobenzotrifluoride is the major product. The key masses of the MS are highlighted. Full MS data are available in the supplemental material (Fig. S8). ^19^F- and ^1^H-NMR data are provided in Materials and Methods.

P. putida F1 wild type and P. putida F39/D, a mutant strain lacking an active dihydrodiol dehydrogenase (49), each was incubated separately with 4-fluorobenzotrifluoride via a vapor bulb. The wild type gave 1.7 mM fluoride and the mutant 0.7 mM fluoride in overnight cultures. To better understand those observations, we first purified the transformation product from P. putida F39/D cultures. The major product was a *cis*-2,3-dihydrodiol, identified by ^1^H-nuclear magnetic resonance (NMR) and ^19^F-NMR, as presented in Materials and Methods and Fig. S7. The four fluorine atoms remained in this product. ^1^H-NMR was compared to the *cis*-2,3-dihydrodiol produced by toluene dioxygenase in Pseudomonas putida UV4 in Boyd et al. (48), which confirmed the major product to be 4-fluorobenzotrifluoride-2,3-dihydrodiol (i.e, 1,2-dihydroxy-3-trifluoromethyl-6-fluorocyclohexa-3,5-diene) (48). Next, we extracted transformation products from the wild-type culture and used bis-trimethylsilane (bis-TMS) as a derivatizing reagent. Gas chromatography-mass spectrometry (GC-MS) revealed a range of products (Fig. 4). We observed 4-fluorobenzotrifluoride-2,3-dihydrodiol, underivatized and partially derivatized, a phenol and the major product, 4-fluoro-(trifluoromethyl)catechol (i.e, 3-trifluoromethyl-6-fluoro-1,2-benzenediol). These are the stable products that still contain fluorine. Note that in Fig. 4 the scale is 20-fold higher for the catechol, which we estimate to represent >90% of the recovered products.

**FIG 5:**
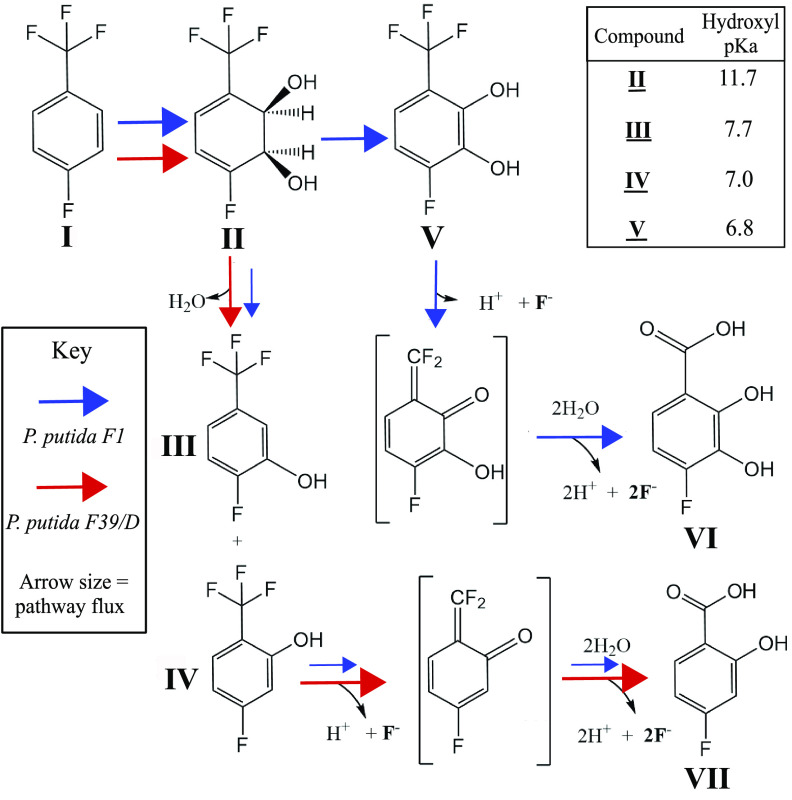
Schematic showing the oxygenation and defluorination of 4-fluorobenzotrifluoride. Arrows show pathway differences between P. putida F1 and P. putida F39/D, with the size of the arrows illustrating the relative magnitude of the products. In the top row, the products II and V are formed by toluene dioxygenase and *cis*-dihydrodiol dehydrogenase, respectively. Compounds III and IV derive from spontaneous dehydration of compound II. Compounds VI and VII arise from V and IV, respectively, via the relatively low pK_a_ phenolic groups undergoing deprotonation and fluoride elimination to form the quinone methide intermediates shown in brackets, following established chemistry (51, 55). The pK_a_s of the hydroxyl groups are indicated in the inset at the upper right, predicted using SciFinder (54). The pK_a_s for dihydroxy compounds are for the hydroxyl group *ortho-* to the trifluoromethyl group.

P. putida F39/D also produced phenolic products, with the hydroxyl *ortho-* or *meta-* to the trifluoromethyl group, consistent with many studies showing that *cis*-dihydrodiols can readily undergo dehydration (19, 50, 51) (Fig. 5). Further experiments were conducted with synthetic standards of each phenol. The *meta*-phenol (III) was quite stable in growth medium and buffers at different pH values. The *ortho*-phenol (IV) is not stable and readily released fluoride into growth medium or buffer solutions. This explains fluoride release by P. putida F39/D. Extraction of the medium and subsequent GC-MS, ^1^H-NMR, and ^19^F-NMR spectroscopy identified 4-fluorosalicylate (VII) (Fig. S9). This compound, VII in Fig. 5, was shown experimentally to be derived from spontaneous defluorination of the *ortho*-phenol (IV). Defluorination of the *ortho*-phenol was rapid in the growth medium and accelerated at pH values above the pK_a_ value of 7.0 (Fig. 5). This is consistent with the defluorination being driven by the deprotonation of the phenolic hydroxyl group with the intermediate formation of a difluoro-quinone methide. This is shown by the middle-bracketed compound in Fig. 5. Base-catalyzed difluoro-quinone methide formation has been demonstrated with 2-(trifluoromethyl)phenol (52), and the additional fluorine substituent on the ring, in the compound studied here, lowers the pK_a_. Groups that lower the hydroxyl pK_a_ will increase the rate of defluorination, as reported in numerous studies with other (trifluoromethyl)phenols (52, 53).

Additional experiments were done with E. coli pDTG 602, a recombinant strain that only expresses toluene dioxygenase (TDO) and dihydrodiol dehydrogenase (TodD) and accumulates catechols (54), which was grown and incubated with 4-fluorobenzotrifluoride. After incubation, 4-fluoro-(trifluoromethyl)catechol (V) was extracted and identified by GC-MS (Fig. 4) and via ^1^H-NMR and ^19^F-NMR (Fig. S10). Compound V is predicted to have the lowest pK_a_ of 6.8 (55) and, thus, was expected to undergo fluoride elimination readily. The defluorination of 4-fluoro-(trifluoromethyl)catechol is proposed to proceed via the quinone methide shown in brackets, followed by hydrolysis to give complete defluorination. The quinone methide intermediate has been discussed previously by Kiel and Engesser (56) with 3-trifluoromethyl catechol. In our experiments, the defluorination is predicted to be even more facile due to the additional fluorine substituent. This explains the greater amount of fluoride observed with wild-type P. putida F1 than with the mutant P. putida F39/D. Millimolar levels of fluoride release were detected in many experiments. However, stoichiometric calculations were not possible because 4-fluorobenzotrifluoride is highly volatile, additions cannot be controlled, and the substrate was by necessity added via a vapor bulb.

4-Fluorosalicylate or 4-fluoro-2,3-dihydroxy benzoic acid (VI) did not undergo further metabolism that we could discern. The ring cleavage product is reported to absorb strongly in the visible region, 375 nm, with an extinction coefficient of 31,600 cm^−1^ M^−1^ (57). There was no evidence for this product or its further metabolism to trifluoroacetic acid. Moreover, we observed a similar product profile with P. putida F1 when we used E. coli(pDTG602) that lacks enzymes to transform the catechols. Previous studies with trifluoromethylbenzoates (58) and other electron-withdrawing substituents (59) have also observed no or very low activity of ring cleavage dioxygenases with the respective substrates. Other studies have observed ring cleavage with trifluoromethyl catechols (60, 61), suggesting that catechol 2,3-dioxygenases show variation in their reactivity. It is also possible that the extra fluorine substituent *para-* to the trifluoromethyl group negates ring cleavage. Regardless of the explanation, it is the block of further metabolism and accumulation of the trifluoromethyl catechol and *ortho*-phenol that leads to extensive defluorination.

## DISCUSSION

Numerous colorimetric methods are known to determine fluoride in organic solvents and pure water, but this study overcame interferences in an aqueous method to apply rapid microwell determinations for studying microbiological defluorination reactions *in vitro* and *in vivo*.

Currently, there is an increasing interest in determining inorganic and organic fluorine in many different types of samples (62). Thousands of organofluorine compounds have been developed, and they are becoming more heavily regulated (63), so new analytical methodologies are needed (24, 64). There is also a need for determining inorganic fluorine, principally fluoride, in natural waters and in municipal drinking waters where fluoride is added for cavity prevention (65). To meet that need, several analytical methods for determining fluoride in water were developed. The most pervasive of the methods was based on color formation from fluoride binding to an alizarin dye complexed with a rare earth metal: cerium, lanthanum, or praseodymium (36). They subsequently used cerium as the fluoride binding metal (35). Variations of the method have been used to determine fluoride after extractions from silicate rocks (66, 67).

More recently, there has been increased interest in determining fluoride relevant to biological systems and processes. Fluoride is toxic to microorganisms, and many strains use a fluoride-sensitive riboswitch to sense the anion and induce a resistance response (42). A fluoride riboswitch was adapted subsequently to detect fluoride intracellularly (68) and extracellularly (69, 70). The latter built on the natural regulatory property of the fluoride riboswitch, in these cases engineered to give a readout of fluoride from the transcriptional product.

Another analytical need is monitoring the biodegradation of fluorinated compounds. Many so-called PFAS compounds and their potential metabolites are determined daily by academic, industry, and government scientists using expensive extraction, chromatography, and mass spectrometry methods. However, fluoride determination is relatively inexpensive and can be applied to any of the thousands of fluorinated organic compounds under study. In laboratory microbiology experiments, there is a need for determining fluoride in solid and/or liquid media or in buffers for resting cells or enzyme reactions. In the present study, we developed a more versatile method that could be used directly with certain media and buffers. Building on past work using a sensitive metal-based fluoride binding assay (35, 36), we determined (i) the metal showing most reproducibility for our purposes was lanthanum, (ii) the signal formed within minutes and was stable in certain buffer solutions, and (iii) a range of buffers, media, and biological additives did not interfere with fluoride determination.

We chose to test the microtiter well method developed here by screening Pseudomonas putida F1 against a library of 63 fluorinated aromatic compounds and comparing the results to measurements made in larger volumes using a fluoride electrode. P. putida F1 was chosen because it previously showed significant rates of defluorination with 2,2-difluoro-1,3-benzodioxole (19), catalyzed by toluene dioxygenase, and this organism is known to express other aromatic-metabolizing enzymes (71, 72). As such, it was considered likely to show defluorination with multiple substrates. The library choice also reflects that a significant number of fluorinated aromatic compounds can be purchased commercially and economically. The combination of organism and substrates provided for multiple opportunities to see what could interfere with the screen and for a reasonable numerical test of sensitivity and selectivity of the method.

Following the significant extent of defluorination of these compounds here, a deeper investigation served to better explain the environmental fate of trifluoromethylphenyl compounds. While oxidative defluorination has been demonstrated previously (29, 73, 74), the mechanism of defluorination of 4-fluorobenzotrifluoride was not immediately obvious. Moreover, the trifluoromethylphenyl moiety has become common in drugs and agricultural chemicals, with the goal of increasing effective lifetime by inhibiting microbial degradation (9, 75). 4-Fluorobenzotrifluoride underwent only partial transformation via the toluene biodegradative (Tod) pathway, and this was the key to the large extent of defluorination observed. Indeed, it was determined here that the metabolic weakness of the trifluoromethyl group derives from the strongly electron-withdrawing propensity of the three fluorine substituents to drive the formation of a *gem*-difluorovinyl (difluoromethide) functionality via the metabolic incorporation of electron-donating groups *ortho*- or *para*- to the trifluoromethyl moiety (Fig. 5). This has been described previously for the hydroxylation of α,α,α-trifluorotoluene (benzotrifluoride) (56). 4-Fluorobenzotrifluoride is predicted to defluorinate faster than benzotrifluoride due to the lower pK_a_ of the phenol group for the former compound, and this was indicated here via the color screening method.

In conclusion, the development of a rapid screening method has facilitated multiple discoveries for new defluorination reactions, including a cryptic ability to degrade trifluoromethylphenyl groups, a very significant functionality in new pharmaceutical and agricultural chemicals. Rapid screening by visual inspection or machine will allow for greater discovery in, and the engineering of, new defluorination biochemistry.

## MATERIALS AND METHODS

### Chemicals.

Alizarin-3-methyliminodiacetic acid was purchased from Cayman Chemical. Lanthanum(III) nitrate hexahydrate, cerium(III) nitrate hexahydrate, neodymium(III) nitrate hexahydrate, and praseodymium(III) nitrate hexahydrate, at 99.99% purity, were from Sigma-Aldrich. Sodium acetate used for the buffer was from J. T. Baker and was 99% pure. Glacial acetic acid was from Fisher Scientific. Acetone from Fisher Scientific and ethyl acetate and methyl-*tert-*butyl-ether (MTBE) from Sigma-Aldrich were high-performance liquid chromatography (HPLC) grade and >99% pure. Fluoroatrazine was synthesized (76), and ^1^H-NMR analysis indicated a purity of ∼90%. 4-Fluorobenzotrifluoride was purchased from Oakwood Chemical. GC-MS and NMR data show ∼98% purity of this compound. Standard 5-fluoro-2-(trifluoromethyl)phenol and 2-fluoro-5-(trifluoromethyl)phenol were both purchased from Sigma-Aldrich at ∼97% purity. l-Arginine, used for growth, was from Calbiochem. Isopropyl-β-d-thiogalactopyranoside (IPTG) was obtained from Gold Bio. Toluene, used for specified cell growth and induction, was from Fisher Chemical and had 99% purity. *N*,*O*-Bis(trimethylsilyl)trifluoroacetamide was purchased from Fluka Analytical and had a purity of 99%. CDCl_3_ and CD_3_CN were obtained from Cambridge Isotope Laboratories, Inc., both at 99% purity.

### Screen development.

The method was developed by testing different alizarin-3-methyliminodiacetic acid complexone (alizarin) concentrations, different rare earth metals [lanthanum(III) nitrate hexahydrate, neodymium(III) nitrate hexahydrate, praseodymium(III) nitrate hexahydrate, and cerium(III) nitrate hexahydrate], and various acetate buffer pHs and concentrations. The acetate buffer was prepared as done in Belcher and West (36) using sodium acetate and glacial acetic acid. Stock solutions of alizarin and metals were each prepared in water to concentrations of 500 μM. Individual stock solutions were kept in the dark and found to be stable for at least 6 months. For use in the assay, alizarin and acetate buffer were mixed first and then the metal was added. This solution was then added to samples containing fluoride, followed by the addition of the organic solvent acetone. The complexone solution of alizarin, metal, and acetate buffer was made fresh before each experiment and was stable for up to 2 h.

First, each metal was tested with various concentrations of fluoride to evaluate sensitivity. It was determined that lanthanum(III) was best for our applications. Concentration and pH were also tested. To determine ideal pH of the reaction, the pH of the acetate buffer was modified using hydrochloric acid or sodium hydroxide. pH values of 2.8, 3.3, 4.5, 5.0, 5.5, and 6.0 were all tested. The pH range of 5 to 5.5 was found to be the most sensitive for detecting fluoride in the screen. To test concentrations, reagents were combined in various ratios of lanthanum to alizarin (1:1, 2:1, 1:2, and 2× and 3× the concentration previously used [36]) and reacted with a given concentration of fluoride in a 96-well plate for 5 to 10 min at room temperature, at which point wells of the plate were read using a Molecular Devices SpectraMax 384plus plate reader. Samples were scanned from 350 nm to 700 nm. Optimal sensitivity was determined by a linear absorbance pattern at 620 nm.

Ideal final concentrations of each compound were found to be 50 μM alizarin-3-methyliminodiacetic acid, 50 μM lanthanum, 84 mM acetate buffer (three times the concentration used by Belcher and West [36]), and 25% (vol/vol) acetone. Higher concentrations of fluoride were visually detected after 5 min. However, low concentrations of fluoride were allowed to react for 30 to 60 min before visual detection. The reaction color is stable for up to 3 h.

Serial dilutions to observe interferences in screen sensitivity were performed. A known concentration of fluoride was added to the first well in a series. The following wells would be diluted with either the medium of interest or water. Half of the volume would be taken from the initial well and then diluted subsequently 1:2 into the other wells. Screen components were combined and added to each well at concentrations described above.

In visual determination of fluoride release in the color screen, qualitative observations could be made. High fluoride release was a dark purple well, designated ++. Low fluoride release was a light purple well, designated +. The dark and light colors were defined quantitatively using the absorbance at 620 nm divided by the absorbance at 530 nm from standards. Wells defined as having darker color (designated ++) have a 620/530 ratio above 0.7. Wells with lighter purple color (designated +) showed a 620/530 value of <0.7 (Fig. 2). Red wells, or no fluoride release, showed values of <0.6. Interferences were identified in wells that were an altered color or intensity, such as dark brown, gray, yellow, or light pink (see Fig. S2 in the supplemental material), and do not have a specified ratio value. The results were found to correlate with fluoride determinations made with a fluoride-specific electrode as shown in Results.

### UV-Vis.

Samples were placed in a quartz cuvette, and measurements were collected on a Cary 3500 double-beam UV-visible (UV-Vis) spectrometer containing a Xenon flash lamp from Agilent. Absorbance as a function of wavelength was recorded from 350 nm to 700 nm. Samples were tested at pH 5.2 and used with the optimum reagent concentrations described above. The Results section shows the samples: (i) deionized (DI) water; (ii) 2 mM sodium fluoride in DI water; and (iii) 2 mM sodium fluoride in mineral salts basal medium (MSB) that contains 30 mM phosphate.

### Interfering and noninterfering substances.

The fluoride screen has been made amenable to microbiological experiments here by determining buffers and media that show noninterference with color development. Buffer, media, and other components in normal aqueous solution were tested at concentrations given in Table 1. To each solution, 2 mM fluoride was added and then the standard screen conditions were used as described above. No interference was defined as matching the color intensity of the control and having no alterations to color. Moderate interference was defined as a slightly lighter purple (difference in intensity) sample with no drastic color change. Strong interference is described as a sample with an obviously different color (i.e., red, orange, or brown) or intensity (i.e., transparent). The presence of a moderately interfering compound may reduce the sensitivity of the screen for lower concentrations of fluoride, while a strong interfering compound would make it virtually impossible to detect the presence of fluoride, even at high fluoride concentrations.

### Bacterial strains and growth conditions.

Pseudomonas putida F1 was maintained on Luria-Bertani (LB) plates, and the strain was grown in liquid on minimal MSB (77) and toluene in a vapor bulb, as previously described (78), at 28°C. Pseudomonas putida F39/D was also maintained on LB plates and grown on MSB and 0.2% (wt/vol) arginine at 28°C. Escherichia coli pDTG 602 was grown on LB-ampicillin (100 μg/mL) as described previously (19).

### Kinetic assay for fluoroatrazine defluorination.

TrzN enzyme was expressed and purified with small variations to a previously described protocol (40). One liter of Escherichia coli pAG TrzN was grown and induced as in Shapir et al. (79). After growth, the cells were centrifuged, resuspended in buffer, and passed through a chilled French press cell at 140 MPa to lyse the cells. The cell crude extract was centrifuged at 16,000 rpm for 1 h. The centrifuged extract was filtered through a 0.45-μm filter. A 5-mL nickel column was used to purify the His-tagged TzN (40) protein from lysate. The column was washed with water and 20 mM Tris with 10% glycerol (buffer A). The sample was then placed on the column with an additional 3 mL of buffer A. Protein was eluted from the column using a second buffer containing 20 mM Tris, 500 mM imidazole, and 10% glycerol (buffer B). Fractions containing purified TrzN were then buffer exchanged using 30-kDa Amicon Ultra-15 cellulose centrifugal filters into 20 mM HEPES buffer containing 10% glycerol. Buffer was exchanged until the imidazole concentration was less than 60 μM. It was important that the concentration of Tris and imidazole be reduced, since they were observed to interfere with the color screen.

The color screen was used to monitor the course of fluoride release from fluoroatrazine in separate reactions catalyzed by NaOH and heat or TrzN enzyme. For the base-catalyzed reaction, fluoroatrazine was added to make 2 mM in 50 mL water adjusted to pH 11 with NaOH and at 70°C. Aliquots were taken in microtiter plates at fixed time points for up to 4 h and immediately frozen. At the conclusion of the time course the aliquots were thawed and assayed by the standard alizarin-La reaction mixture. Enzymatic defluorination was carried out with 250 nM TrzN and 2 mM fluoroatrazine in 20 mM HEPES (pH 7.0). The reaction was sampled and assayed as described above for the base- and heat-catalyzed defluorination mixture. Note that fluoroatrazine is not soluble in water/buffer at a 2 mM concentration. The excess solid material was left to float on top of the buffer and solubility increased as the reaction proceeded.

### Rapid microwell screening of defluorination catalyzed by Pseudomonas putida F1.

P. putida F1 was grown in a 150-mL culture in MSB medium with a vapor bulb containing toluene to an optical density at 600 nm (OD_600_) of 0.4. The cells were pelleted and resuspended in 75 mL of 20 mM HEPES, and 1-mL aliquots were transferred into individual wells of a 96-deep-well plate. A 40 mM chemical stock in 20 mM HEPES was added in 100-μL aliquots to each well. Although many of the fluorinated chemicals are not soluble at 40 mM in HEPES, each stock solution was vortexed and mixed thoroughly before use in each assay. Many studies use substrates above the solubility limit in P. putida F1 experiments, and the organism continually metabolizes the chemicals as they dissolve. The plate was incubated overnight at 28°C on an Eppendorf Thermomixer R plate shaker at 400 rpm. Prior to fluoride determination with the color screen, the cells were pelleted in the deep-well plate using a swinging-bucket rotor at 1,000 rpm for 20 min. Supernatant solutions (100 μL) were transferred to a flat-bottom 96-well plate and screened as described above for screen development.

### Fluoride detection with fluoride electrode.

Using 1 mL of sample supernatant, an ion-plus SureFlow fluoride electrode from Thermo Scientific (Coon Rapids, Minnesota) was used as in Bygd et al. (19).

### Generation and analysis of intermediates and products from tetrafluorinated compounds.

Biological transformation of 4-fluorobenzotrifluoride was conducted with Pseudomonas putida F39/D. The strain was grown overnight on MSB and 0.2% (wt/vol) arginine and was used to inoculate cultures grown in MSB containing 0.2% (wt/vol) arginine and toluene in a vapor bulb. The cultures were grown to early log phase at 28°C, and then the toluene was replaced with 4-fluorobenzotrifluoride and incubated for an additional 2 h. Subsequently, the cells were pelleted and 1 mL of cleared medium was frozen for later GC analysis. The remaining supernatant was extracted in a separatory funnel with equal volumes of ethyl acetate. The extract was dried with anhydrous magnesium sulfate and rotary evaporated under vacuum. Once dry, the crystallized sample was dissolved in deuterated chloroform. ^1^H- and ^19^F-NMR both were performed as described below. The ^19^F-NMR showed the dihydrodiol product from 4-fluorobenzotrifluoride only. The ^1^H-NMR showed largely the dihydrodiol product from 4-fluorobenzotrifluoride with some contaminating toluene dihydrodiol. Both of those ^1^H-NMR experiments have been described previously, so they were readily distinguishable (48).

Biological transformation of 4-fluorobenzotrifluoride was also conducted with Escherichia coli pDTG 602, which was grown to mid-log phase on LB with ampicillin (100 μg/mL) at 37°C. Subsequently, the cells were pelleted and resuspended in MSB (at an OD_600_ of 0.65) and 0.2% glucose and supplemented with 1 mM IPTG for induction (54). After 1 h at 30°C, a vapor bulb containing 4-fluorobenzotrifluoride was added to the culture and incubated an additional 2 h. After 2 h, the medium appeared purple and was centrifuged at 10,000 rpm for 10 min. The cell-free supernatant was extracted and handled as described above for P. putida F39/D.

An abiotic transformation was conducted by adding 8 mM 5-fluoro-2-(trifluoromethyl)phenol to MSB (pH 9.3) with shaking at 200 rpm at 28°C overnight. Fluoride was measured using a fluoride electrode. Strong acid was added to adjust the pH of the medium to pH 1.9. The sample was extracted in a separatory funnel with equal volumes of ethyl acetate and dissolved in deuterated chloroform, as described above. The sample was poorly soluble in chloroform, so it was filtered through cotton. A small amount of the product remaining on the cotton filter was then placed in fresh CDCl_3_ and used for NMR, using trimethylsilane (TMS) as a reference for ^1^H-NMR.

### Analytical methods.

GC-MS was conducted as in Bygd et al. (19), except that the GC oven temperature started at 40°C instead of 50°C. Standard solutions of 5-fluoro-2-(trifluoromethyl)phenol and 2-fluoro-5-(trifluoromethyl)phenol were prepared in methyl-*t-*butyl ether (MTBE). One microliter of derivatizing agent, *N*,*O*-bis(trimethylsilyl) trifluoroacetamide, was added to each extracted sample or standard. Product ion spectra were identified in positive ion mode on an HP6890 gas chromatograph with an HP5973 MS detector.

NMR was performed using a Varian INOVA 400-MHz NMR spectrometer. For both ^1^H-NMR and ^19^F-NMR, deuterated chloroform (CDCl_3_) was the solvent. TMS and trichlorofluoromethane (F11) served as reference for ^1^H-NMR and ^19^F-NMR, respectively. Extracts from bacterial or abiotic incubations were obtained as described previously.

The product extracted from incubation of P. putida F39/D with 4-fluorobenzotrifluoride was analyzed: ^1^H-NMR (400 MHz, CDCl_3_), 4-fluorobenzotrifluoride-2,3-dihydrodiol; 4.53 ppm (apparent t, *J *= 5.5 Hz and 6.0 Hz), 4.62 ppm (d, *J *= 6.44 Hz), 5.65 ppm (dd, *J *= 6.6 Hz and 9.2 Hz) 6.55 ppm (m), ^19^F-NMR (400 MHz, CDCl_3_), −66.04 ppm (s), −109.33 ppm (m) (Fig. S7).

The products extracted from the 5-fluoro-2-(trifluoromethyl)phenol incubation in MSB medium were analyzed: ^1^H-NMR (400 MHz, CDCl_3_), 4-fluorosalicylate; 1.25 ppm (s), 6.7 ppm (apparent dt, *J *= 2.5 Hz and 3.3 Hz), 7.95 ppm (dd, *J *= 6.4 Hz and 8.8 Hz), 10.65 ppm (d, *J *= 1.6 Hz), ^19^F-NMR (400 MHz, CDCl_3_), 4-fluorosalicylate; −99.58 ppm (m), 5-fluoro-2-(trifluoromethyl)phenol; −67.71 ppm (s) (Fig. S9).

The products extracted from incubation of E. coli pDTG 602 with 4-fluorobenzotrifluoride were analyzed: ^1^H-NMR (400 MHz, CDCl_3_), 4-fluoro-(trifluoromethyl)catechol; 6.75 ppm (apparent t, *J *= 9.0 Hz and 9.6 Hz), 7.09 ppm (dd, *J *= 6.0 Hz and 9.0 Hz), and additional minor product was identified as 4-fluorobenzotrifluoride-2,3-dihydrodiol, as seen in spectra described above for ^1^H-NMR. Additional minor peaks in this spectrum were not identified. ^19^F-NMR (400 MHz, CDCl_3_), 4-fluoro-(trifluoromethyl)catechol; −61.91 ppm (s), −132.83 ppm (bs), a second minor product in this spectra was identified as 4-fluorobenzotrifluoride-2,3-dihydrodiol, as described above for ^19^F-NMR (Fig. S10).
