# Supplementary material for: Microwell Fluoride Screen for Chemical, Enzymatic, and Cellular Reactions Reveals Latent Microbial Defluorination Capacity for −CF3 Groups
Source: Appl Environ Microbiol. 2022 Jun 21;88(9):e00288-22. doi: 10.1128/aem.00288-22 (PMC9088286; doi:10.1128/aem.00288-22)
Supplement: Supplemental file 1 — Fig. S1 to S10. Download aem.00288-22-s0001.pdf, PDF file, 2.7 MB [file aem.00288-22-s0001.pdf]

SUPPLEMENTAL MATERIAL

Microwell Fluoride Screen for Chemical, Enzymatic, and Cellular Reactions Reveals Latent Microbial Defluorination Capacity for -CF_3 Groups

Madison D. Bygd[1,2], Kelly G. Aukema[2,3], Jack E. Richman[2,3], Lawrence P. Wackett*[1,2,3]

[1] Microbial Engineering, University of Minnesota

[2] Biotechnology Institute, University of Minnesota

[3] Biochemistry, Molecular Biology and Biophysics, University of Minnesota

Table of contents

Fig. S1. Scan of samples over 620 nm and the linear absorbance of samples 0-100 µM

Fig. S2. Spectrophotometric scans of the alizarin-lanthanum complex in varying pH's and an assay plate of fluoride in various buffers and media

Fig. S3. Serial dilution of samples taken over time during defluorination of fluoroatrazine in chemical and enzymatic reactions

Fig. S4. Color assay in 96 well plate of supernatant from *P.putida* F1 after incubation with full fluorinated chemical compound library and corresponding key

Fig. S5. Plot of fluoride electrode values above and near 30 µM

Fig. S6. All chemicals tested in the library with corresponding electrode determination

Fig. S7. ^{1}H-NMR and ^{19}F-NMR of the *cis*-dihydrodiol produced by *P. putida* F39/D with 4-fluorobenzotrifluoride

Fig. S8. Full mass spectra of GC peaks in main text figure 4

Fig. S9. ^{1}H-NMR, ^{19}F-NMR, and GC-MS of 4-fluorosalicylate derived from spontaneous defluorination of 5-fluoro-2-(trifluoromethyl)phenol

Fig. S10. ^{1}H-NMR and ^{19}F-NMR of the catechol produced by *E.coli* pDTG 602

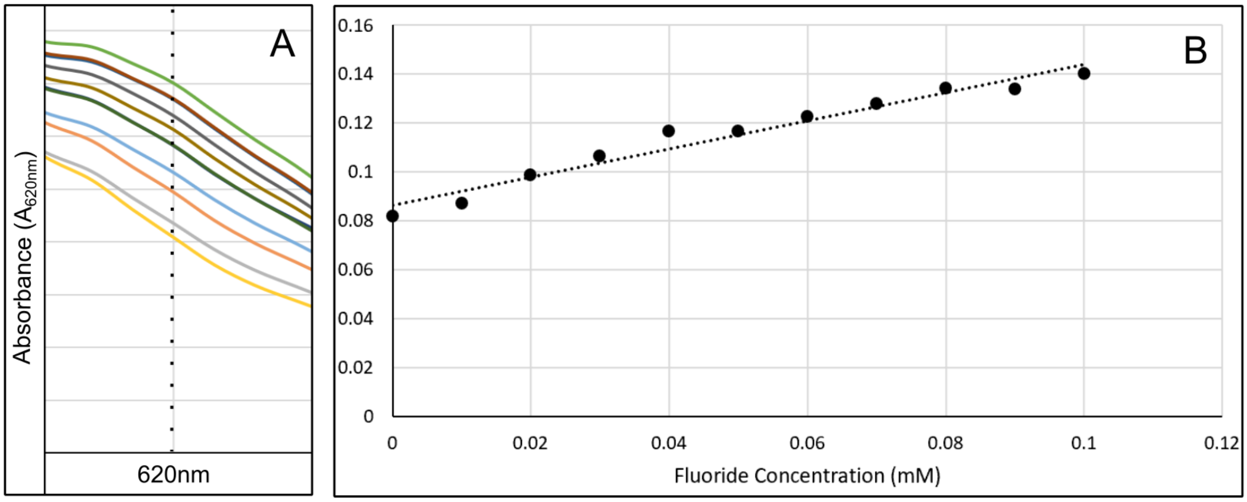

Figure S1. A) Wavelength scan of samples from 0-100 μM fluoride in the alizarin-lanthanum complex over 620nm. This wavelength provided the best linear sample separation, as seen in panel B.

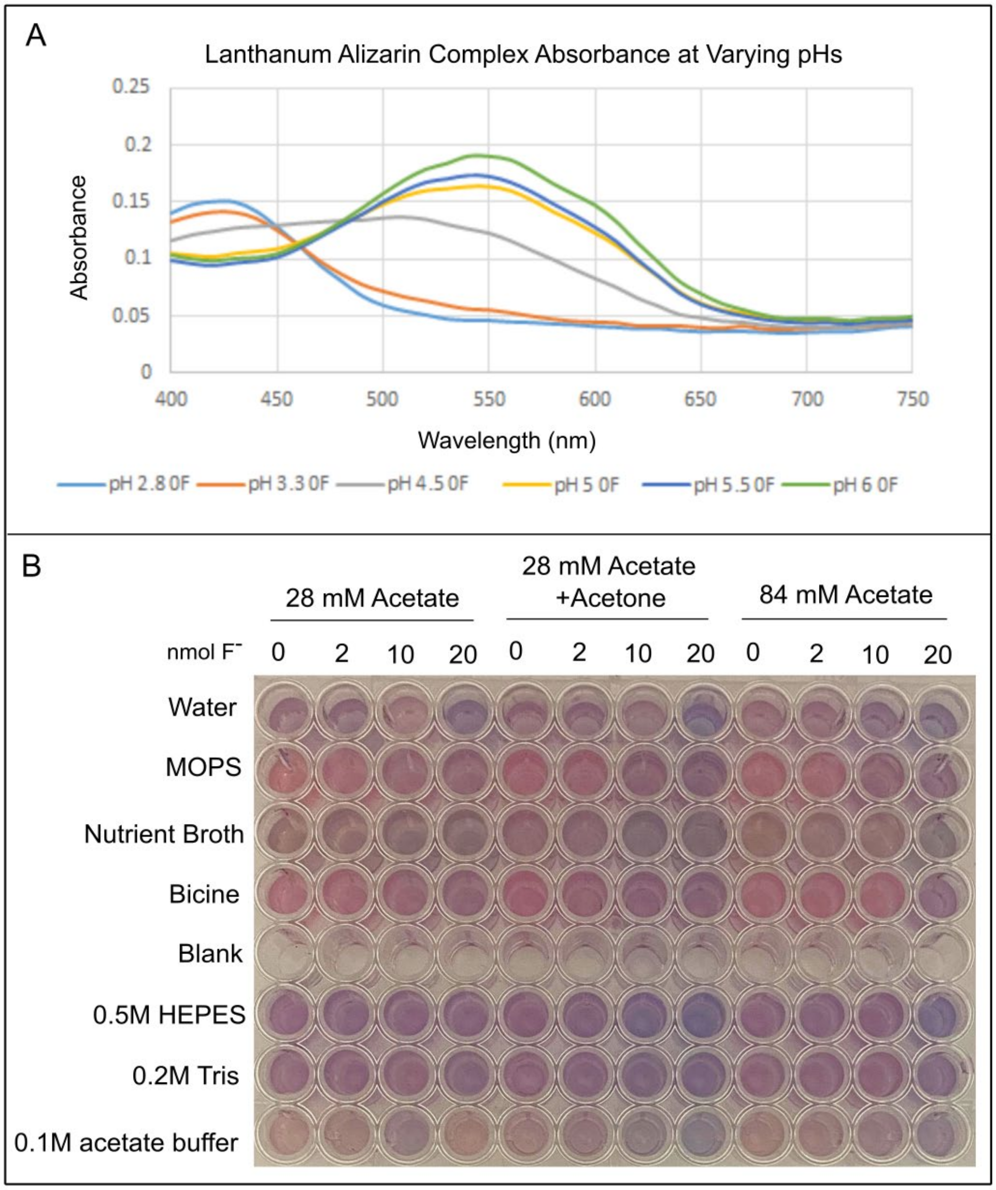

Figure S2. Optimization of conditions for maximizing sensitivity and minimizing interferences for the color assay. A) Spectrophotometric measurements of the difference in Lanthanum-Alizarin absorbance at varying pH's. The samples shown contain no fluoride but illustrate that a higher pH (>5) is necessary for proper complex formation (see main Figure 1). Additional tests (not shown) indicate pH 5.0-5.5 was the most sensitive for detecting fluoride. As previously reported, the sensitivity of the assay with lanthanum was not improved by replacing the metal with cerium, praseodymium or neodymium (1, 2). Spectrophotometric tests were done with the four different rare earth metals with acetone and fluoride (not shown) and indicated comparable absorbance values at 620 nm. Because of previous use and reliability at low fluoride concentrations, lanthanum was used for the remainder of this study. B) Microtiter plate testing various types of media and concentrations of buffer with or without acetone. Level of interference was compared to the well containing only water.

References

1. Belcher R, West T.S. 1961a. A study of the cerium(iii)-alizarin complexan-fluoride reaction. *Talanta* 8**:** 853-862.
2. Belcher R, West, TS. 1961b. A comparative study of some lanthanon chelates of alizarin complexan as reagents for fluoride. *Talanta* 8: 863-870.

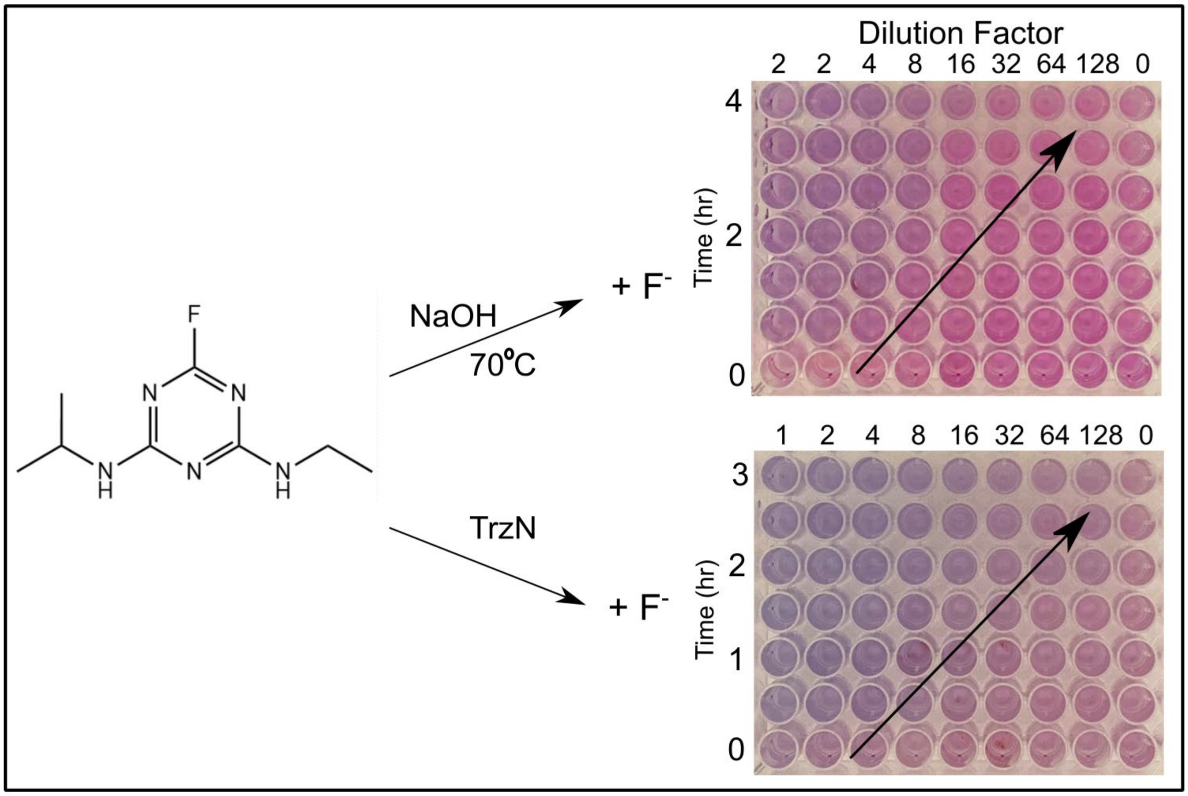

Figure S3. Plated serial dilution over time from chemical (top) and enzymatic (lower) reactions starting with fluoroatrazine and measuring defluorination using the color assay and dilutions to estimate fluoride concentrations increasing over time. Samples were taken from each individual reaction over time and diluted to demonstrate concentration. Purple wells at higher dilutions indicates a higher concentration of fluoride in the sample. The chemical reaction was done in water at pH 11 while the enzymatic reaction was done in 20 mM HEPES buffer (pH 7.3) at 37˚C. There were no interferences with the assay in either reaction.

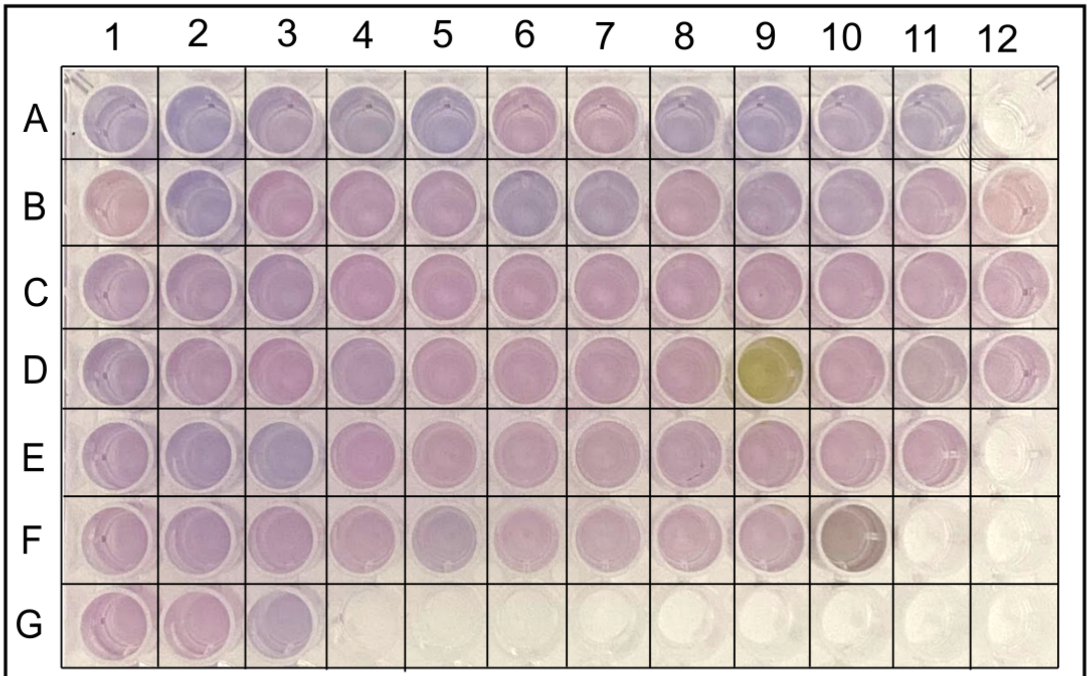

A1	2-fluorotoluene	C1	1,2,3,4-tetrafluorobenzene	E1	4-fluorophenyl acetic acid
A2	3-fluorotoluene	C2	1,2,3,5-tetrafluorobenzene	E2	4-(difluoromethoxy)benzoic acid
A3	4-fluorotoluene	C3	1,2,4,5-tetrafluorobenzene	E3	4-fluoro acetophenone
A4	4-fluorobenzotrifluoride	C4	pentafluorobenzene	E4	4-fluoro-1-methylphenyl acetic acid
A5	ααα-trifluorotoluene	C5	hexafluorobenzene	E5	4-fluorobenzonitrile
A6	5-chloro-2-fluorotoluene	C6	chloropentafluorobenzene	E6	fluoroacetonitrile
A7	2,6-difluorotoluene	C7	perfluorodecalin	E7	3-fluorophenyl hydrazine hydrochloride
A8	2,5-difluorotoluene	C8	3-fluoro-o-xylene	E8	3-fluorouracil
A9	3,5-difluorotoluene	C9	4-fluoro-o-xylene	E9	2,2-difluoro-5-nitro-1,3-benzodioxole
A10	2,3-difluorotoluene	C10	2-fluoro-m-xylene	E10	2,2-difluoro-1,3-benzodioxole-carboxylic acid
A11	2,4-difluorotoluene	C11	4-fluoro-m-xylene	E11	heptafluorobutyramide
B1	2,3,4-trifluorotoluene	C12	5-fluoro-m-xylene	F1	1-(1-bromoethyl)-4-fluorobenzene
B2	2,4,5-trifluorotoluene	D1	(difluoromethoxy)benzene	F2	2-fluoro-4-(propanyl)benzoic acid
B3	2,3,6-trifluorotoluene	D2	(trifluoromethoxy)benzene	F3	4-fluorocumene
B4	2,4,6-trifluorotoluene	D3	4-(trifluoromethoxy)benzene	F4	1-(1-chloroethyl)-4-fluorobenzene
B5	fluorobenzene	D4	phenyl trifluoromethyl sulfide	F5	4-(difluoromethoxy)aniline
B6	1,2-difluorobenzene	D5	1-difluoromethoxy-4-iodobenzene	F6	3-(difluoromethoxy)aniline
B7	1,3-difluorobenzene	D6	4-(difluoromethoxy)bromobenzene	F7	2,4-difluorophenol
B8	1,4-difluorobenzene	D7	1-nitro-4-(trifluoromethoxy)benzene	F8	2,5-difluorophenol
B9	1,2,3-trifluorobenzene	D8	4-(difluoromethoxy)nitrobenzene	F9	2,6-diflluorophenol
B10	1,2,4-trifluorobenzene	D9	1-fluoro-2,4-dinitrobenzene	F10	3-fluorocatechol
B11	1,3,5-trifluorobenzene	D10	2-(difluoromethoxy)nitrobenzene	G1	No compound
B12	2-fluoro-p-xylene	D11	2,4-(dinitrotrifluoromethoxy)benzene	G2	toluene
		D12	3-fluoro-4-methyl acetophenone	G3	2,2-difluoro-1,3-benzodioxole

Figure S4. Representative fluorinated compound screening plate with *Pseudomonas putida* F1. Positive hits for fluoride release are highlighted yellow in the key. Compounds that interfered with the assay by creating an alternative color are highlighted red in the key.

Compound	Electrode calculated concetration (mM)
2-fluorotoluene	0.058
3-fluorotoluene	0.155
4-fluorotoluene	0.034
4-fluorobenzotrifluoride	0.287
aaa-trifluorotoluene	0.638
5-chloro-2-fluorotoluene	0.010
2,6-difluorotoluene	0.008
2,5-difluorotoluene	0.087
3,5-difluorotoluene	0.127
2,3-difluorotoluene	0.088
2,4-difluorotoluene	0.191
2,3,4-trifluorotoluene	0.058
2,4,5-trifluorotoluene	0.119
2,3,6-trifluorotoluene	0.007
2,4,6-trifluorotoluene	0.013
fluorobenzene	0.022
1,2-difluorobenzene	0.123
1,3-difluorobenzene	0.115
1,4-difluorobenzene	0.009
1,2,3-trifluorobenzene	0.061
1,2,4-trifluorobenzene	0.090
1,3,5-trifluorobenzene	0.049
2-fluoro-p-xylene	0.061
1,2,3,4-tetrafluorobenzene	0.032
1,2,3,5-tetrafluorobenzene	0.023
1,2,4,5-tetrafluorobenzene	0.044
pentafluorobenzene	0.005
hexafluorobenzene	0.004
chloropentafluorobenzene	0.007
perfluorodecalin	0.005
3-fluoro-o-xylene	0.005
4-fluoro-o-xylene	0.005
2-fluoro-m-xylene	0.006
4-fluoro-m-xylene	0.009
5-fluoro-m-xylene	0.005

Compound (cont.)	Electrode calculated concetration (mM) (cont.)
(difluoromethoxy)benzene	0.037
(trifluoromethoxy)benzene	0.015
4-(trifluoromethoxy)fluorobenzene	0.009
phenyl trifluoromethyl sulfide	0.029
1-difluoromethoxy-4-iodobenzene	0.006
4-(difluoromethoxy)bromobenzene	0.007
1-nitro-4-(trifluoromethoxy)benzene	0.008
4-(difluoromethoxy)nitrobenzene	0.007
1-fluoro-2,4-dinitrobenzene	0.576
2-(difluoromethoxy)nitrobenzene	0.006
2,4-dinitro(trifluoromethoxy)benzene	0.087
3-fluoro-4-methyl acetophenone	0.002
4-fluorophenylaceticacid	0.019
4-(difluoromethoxy) benzoic acid	0.047
4-fluoro acetophenone	0.083
4-fluoro- 1-methylphenyl acetic acid	0.006
4-fluorobenzonitrile	0.020
fluoroacetonitrile	0.017
3-fluorophenyl hydrazine hydrochloride	0.018
3-fluorouracil	0.008
2,2-difluoro-5-nitro-1,3-benzodioxole	0.013
2,2-difluoro-1,3-benzodioxole-carboxylic acid	0.007
heptafluorobutyramide	0.003
1-(1-bromoethyl)-4-fluorobenzene	0.011
2-fluoro-4-(propanyl)benzoic acid	0.034
4-fluorocumene	0.083
1-(1-chloroethyl)-4-fluorobenzene	0.007
4-(difluoromethoxy)aniline	0.035
3-(difluoromethoxy)aniline	0.027
2,4-difluorophenol	0.027
2,5-difluorophenol	0.017
2,6-diflluorophenol	0.018
3-fluorocatechol	0.286
No compound	0.003
Toluene	0.002
2,2-difluoro-1,3-benzodioxole	0.162

Figure S5. Tested fluorinated compounds and controls with the calculated free fluoride ion concentration after each chemical was incubated with *P.putida* F1. Fluoride ion measurements were taken using the fluoride electrode and a standard curve was used to calculate concentration based on millivolt readings. The structures of compounds with common names are shown below for clarity.

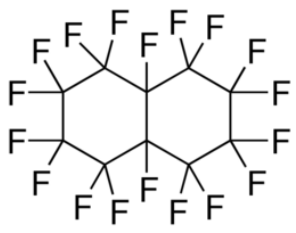

Perfluorodecalin

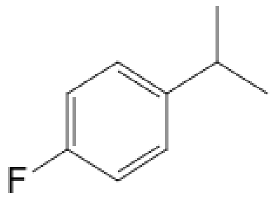

4-Fluorocumene

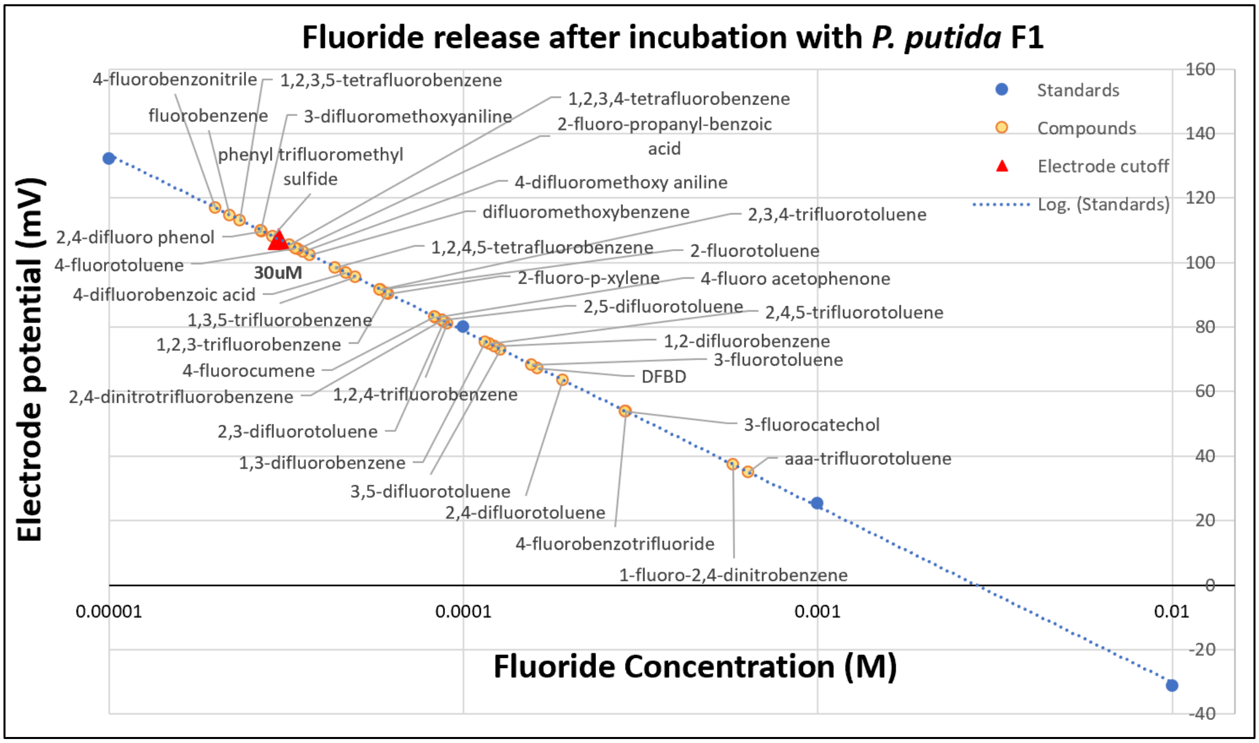

Figure S6. The electrode reading (mV) of supernatant after *P. putida* F1 incubation with each fluorinated chemical plotted against the concentration of fluoride. A standard curve (blue) was created using known concentrations of fluoride (sodium fluoride in 20mM HEPES) and their corresponding electrode readings to calculate the experimental values of fluoride (yellow) released from incubation. The red triangle in the plot indicates the cutoff in which samples were deemed positive hits (>30 µM) or negative hits (<30 µM). The 30 µM point was determined by the approximate visual detection limit of fluoride in the color assay.

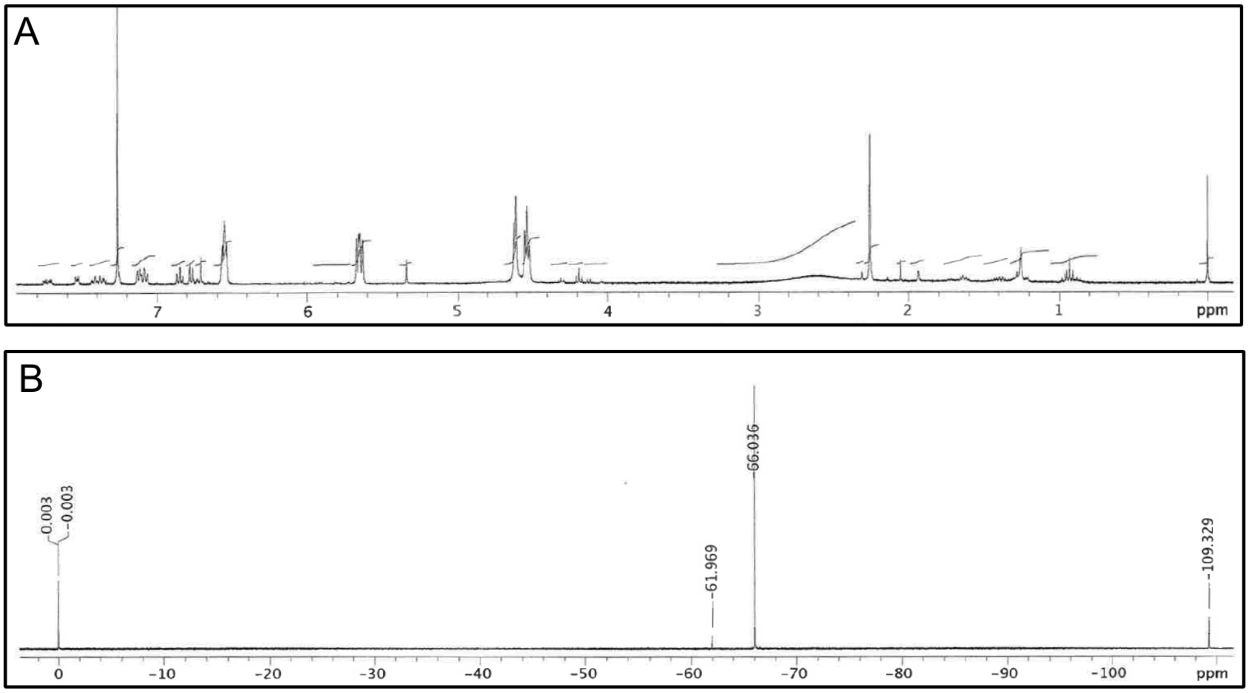

Figure S7. A) ^{1}H-NMR of the products from the extracted supernatant after incubation of *P. putida* F39/D with 4-fluorobenzotrifluoride. B) ^{19}F-NMR of the same extracted supernatant. The major product in these NMR was identified as 4-fluorobenzotrifluoride-2,3-dihydrodiol (i.e, 1,2-dihydroxy-3-trifluoromethyl-6-fluorocyclohexa-3,5-diene). See main text methods for chemical shifts.

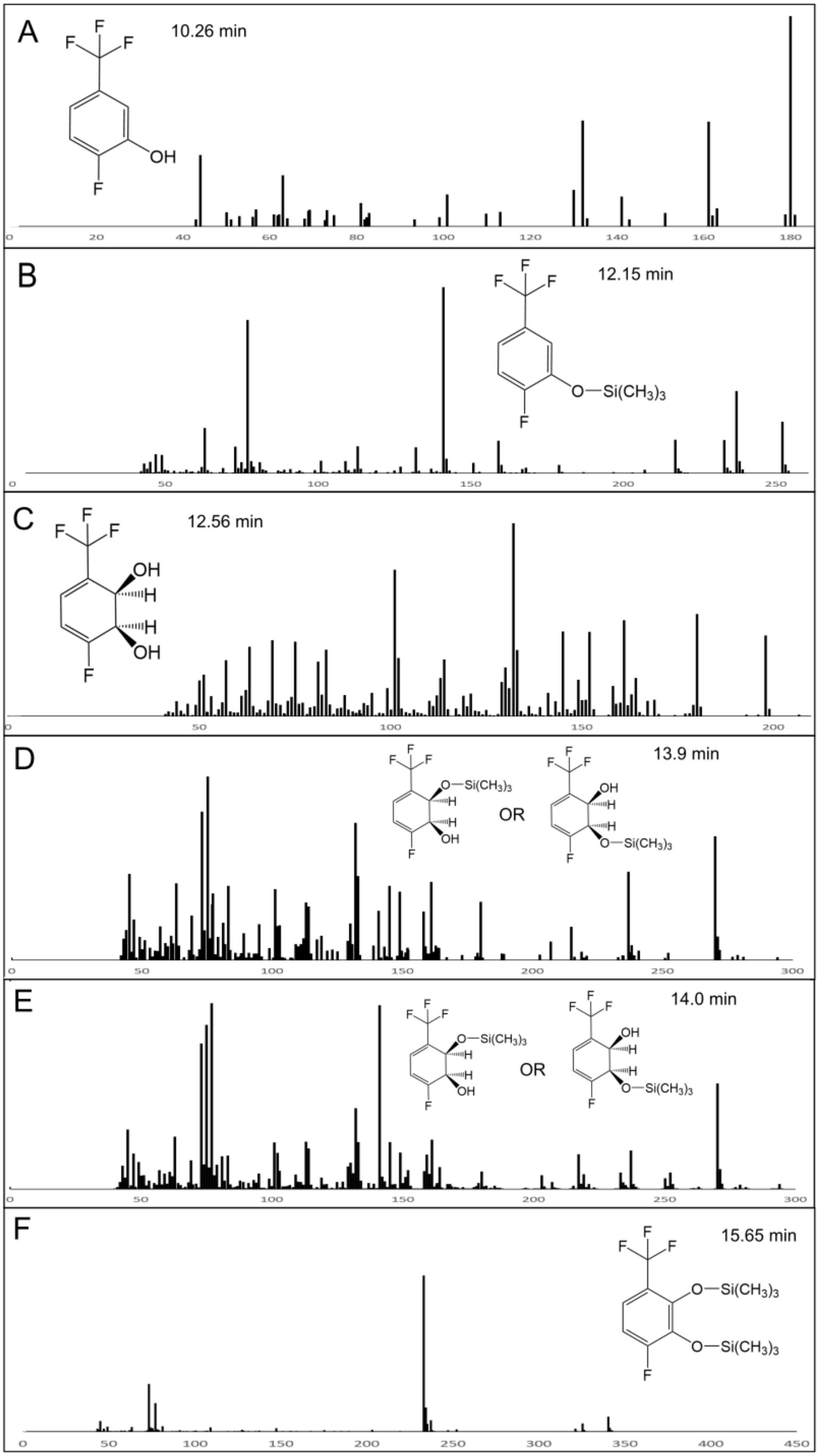

Figure S8. Full mass spectra for the designated peaks in main text figure 4. Fragmentation pattern labeled with corresponding structure and retention time.

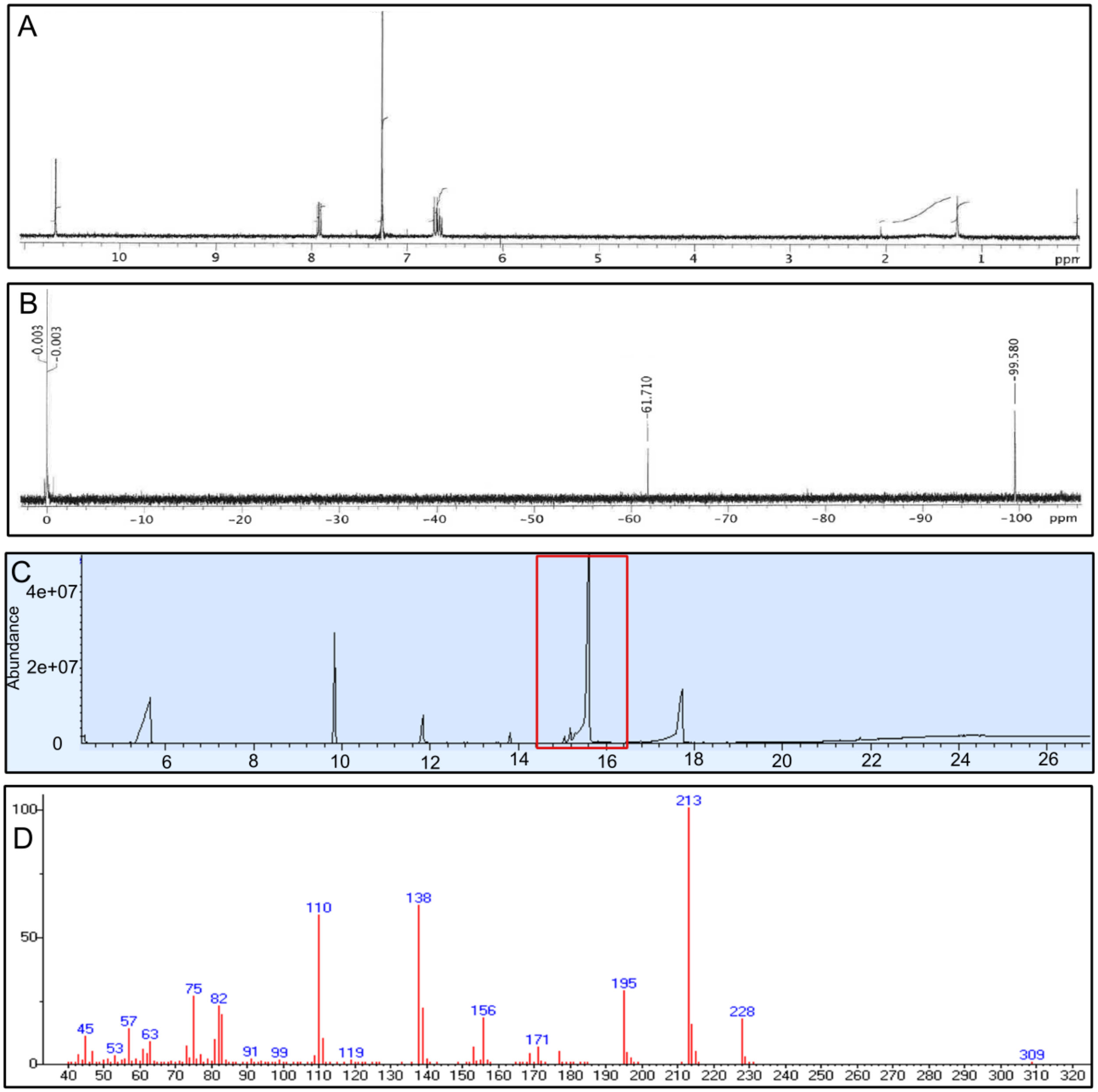

Figure S9. A) ^{1}H-NMR of the products from the extracted media after incubation of 5-fluoro-2-(trifluoromethyl)phenol in MSB overnight. B) ^{19}F-NMR of the same extracted sample. The major product in these NMR was identified as 4-fluorosalicylate. C) GC chromatograph of the extracted product from the spontaneous degradation of 5-fluoro-2-(trifluoromethyl)phenol in MSB. The boxed peak at 15.6 minutes is the most abundant product. D) The relative mass spectra fragmentation pattern of the major peak indicated in panel C shown and the compound was identified to be 4-fluorosalicylate (i.e, 4-fluoro-2-hydroxybenzoic acid).

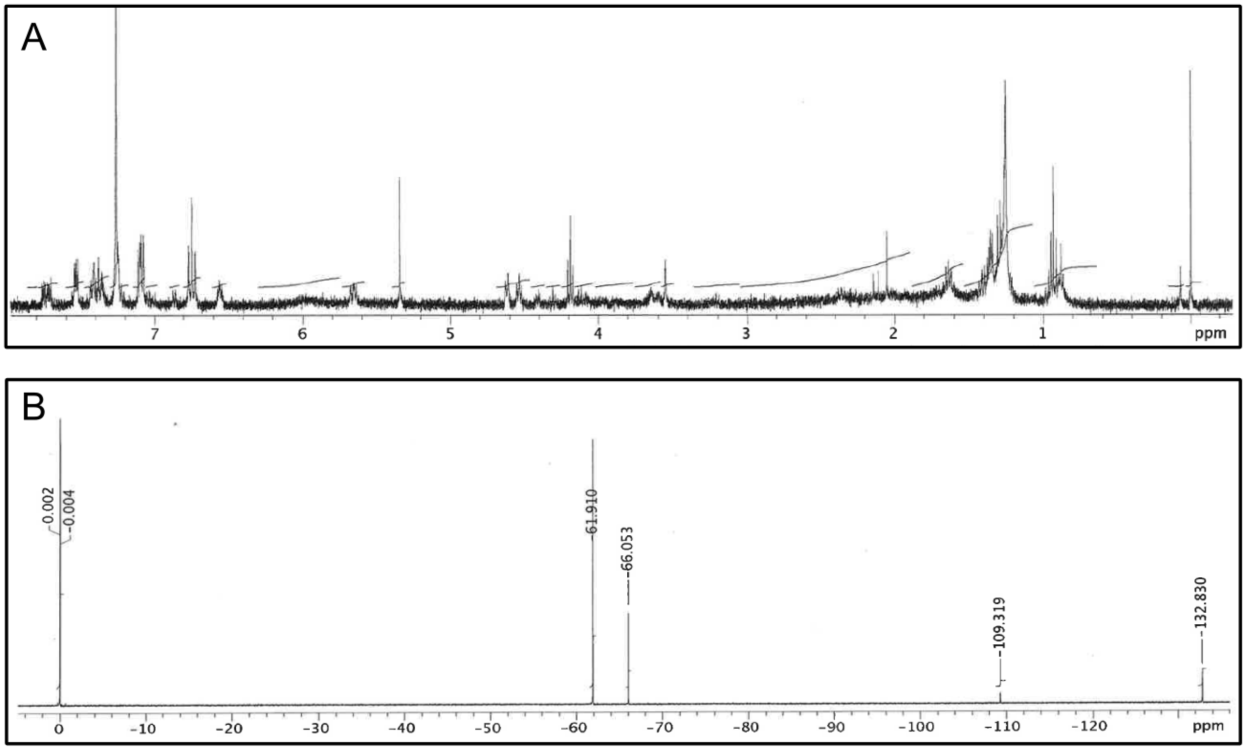

Figure S10. A) ^{1}H-NMR of the products from the extracted material from the culture supernatant after incubation of *E.coli* pDTG 602 with 4-fluorobenzotrifluoride. B) ^{19}F-NMR of the same extracted material. The major product in these NMR was identified as 4-fluoro-(trifluoromethyl)catechol (i.e, 3-trifluoromethyl-6-fluoro-1,2-benzenediol). See main text methods for chemical shifts.
